# Diagnosis and management of headache in children and adolescents: an overview

**DOI:** 10.1186/1824-7288-40-S1-A82

**Published:** 2014-08-11

**Authors:** Vincenzo Belcastro

**Affiliations:** 1 Neurology Unit, S. Anna Hospital, Como, Italy

## Background

Headache is a common complaint in childhood with up to 75% of children reporting a notable headache by the age of 15 years. Pediatric migraine is the most frequent recurrent headache, occurring in up to 28% of older teenagers. Migraine can have a substantial effect on the life of the child, as well as their family, leading to lost school days and withdrawal from social interactions. The prevalence of non-migrainous headache is 10-25% in childhood and adolescence.

## Materials and methods

The distinction of tension-type headache from migraine can be difficult. Although the International Classification of Headache Disorders criteria help, these criteria might be too restrictive to differentiate tension-type headache from migraine without aura in children. A headache diary is a useful method for the differentiation of headache types. The diagnosis of primary headache requires exclusion of secondary headache since many organic disorders can present as tension-type headache because disease-specific features are absent. Fever is the most common cause of benign paroxysmal headache. The basis of diagnosis is a systematic headache history, careful physical and neurological examination, including fundoscopy, and follow-up using a headache diary. If the results of neurological examination are normal in children with frequent headache neuroimaging is not routinely done.

## Results

Several conditions have a comorbid relationship with migraine, such as asthma and allergic disorders, obesity, epilepsy, sleep disorders, and psychological or emotional disorders. The mechanisms by which these comorbid conditions alter the underlying pathophysiology and thus affect the manifestation of migraine are largely unknown. At the biological level, the comorbid disorders might have a common neuropathological pathway, whereas from a behavioural perspective, the difficulty in coping with multiple illnesses might alter the manifestation of the headache. In children, a connection seems possible between tension-type headache and psychosocial stress, psychiatric disorders, muscular stress, or oromandibular dysfunction.

## Conclusions

Most tension-type headache is best managed by primary care. Episodic tension-type headache is self-limiting, but children and their parents generally consult doctors when headache become frequent and are no longer responsive to analgesic. Medication overuse headache can also be a common problem in patients with frequent headache. The treatment of migraine and tension-type headache overlap. Both require acute treatment, either behavioural or pharmaceutical. Preventive pharmaceutical treatment is needed for frequent tension-type headache and migraine.

